# Self-Medication with Antibiotics: Prevalence, Practices and Related Factors among the Pakistani Public

**DOI:** 10.3390/antibiotics11060795

**Published:** 2022-06-12

**Authors:** Adeel Aslam, Che Suraya Zin, Shazia Jamshed, Norny Syafinaz Ab Rahman, Syed Imran Ahmed, Péter Pallós, Márió Gajdács

**Affiliations:** 1 Department of Pharmacy Practice, Kulliyyah of Pharmacy, International Islamic University Malaysia, Kuantan 25200, Malaysia; adeel.aslam224@gmail.com (A.A.); chesuraya@iium.edu.my (C.S.Z.); norny@iium.edu.my (N.S.A.R.); 2 Department of Clinical Pharmacy and Practice, Faculty of Pharmacy, Universiti Sultan Zainal Abidin (UniSZA), Kuala Terengganu 20400, Malaysia; shaziajamshed@unisza.edu.my; 3 School of Pharmacy, College of Science, University of Lincoln, Lincoln LN6 7TS, UK; sia194@yahoo.com; 4 Department of Oral Biology and Experimental Dental Research, Faculty of Dentistry, University of Szeged, 6720 Szeged, Hungary; peterpallos96@gmail.com

**Keywords:** self-medication, antibiotics, antimicrobial resistance, Pakistan, questionnaire, practices, logistic regression

## Abstract

Self-medication with antibiotics (SMA) has become considerably common in developing countries, which is a critical factor for driving antibiotic resistance. Individuals involved in SMA generally do not have adequate knowledge regarding the appropriate use, indications and dosage of these drugs. The objective of the present study was to investigate population SMA practices, knowledge and sociodemographic factors associated with SMA in Islamabad, Pakistan. The study adopted a cross-sectional methodology and data collection was performed through an anonymous, structured and pilot-tested questionnaire, which was interview-administered. Inferential statistics and multivariate logistic regression were performed. Out of 480 participants, 55.6% (*n* = 267) were male with a mean age of 37.1 ± 10.1 years; the total prevalence of SMA was 32.5%. Ciprofloxacin (42.9%) was the most commonly used antibiotic to treat coughs or colds, a runny nose, flu or sore throat, diarrhea or fevers, which were relevant reasons for SMA. Findings from multivariate logistic regression showed that predictors of SMA were: male gender (95% CI: 0.383–1.005), age (95% CI: 0.317–0.953) and highest level of education (95% CI: 0.961–0.649). Despite reasonable access to healthcare facilities, people are still obtaining antibiotics without prescription, bypassing diagnostic and consultative healthcare services. Thus, the government must implement strict healthcare policies to restrict the sale of antibiotics without prescriptions, while at the same time, targeted public awareness campaigns about the proper use of antibiotics are also required.

## 1. Introduction

Self-medication with antibiotics (SMA) is a common practice in both developing and developed countries [1,2,3,4]. The public mostly does not have adequate knowledge about how and when to use antibiotics properly, and due to a lack of professional healthcare supervision—by bypassing diagnostic and consultative healthcare services—they generally misuse prescription antibiotics [5]. Based on the Special Eurobarometer (EBM) Reports of the European Commission, and the World Health Organization (WHO) “Antibiotic resistance: multi-country public awareness survey”, there is a clear association between the knowledge level of the individuals regarding infectious ailments and antimicrobials and the consumption (both via prescriptions and SMA) of antibiotics [6,7]. Studies conducted in Poland [8], Kosovo [9], Ghana [10], Nepal [11] and Pakistan [4] have all reported that non-prescription antibiotics were mainly utilized for inappropriate indications, or for the treatment of symptoms associated with upper respiratory tract infections—such as the common cold and other viral infections—where these drugs are not effective. This inappropriate utilization of antibiotics may lead to several undesirable effects, such as the decreased effectiveness of these drugs, the emergence of difficult-to-treat infections, treatment failure and worsening clinical conditions [5]. At the same time, this utilization has become one of the most critical hallmarks driving antimicrobial resistance (AMR). This trend has been observed throughout the globe and affects health systems at all levels [12]. According to some bleak predictions, by 2050, there will be more than ten million deaths per year directly attributed to AMR; furthermore, it has been estimated that the greatest number of these deaths (~4 million each in Asia and Africa) will burden developing countries [13]. Therefore, there is an urgent need to take action to minimize inappropriate antimicrobial use and the emergence of antimicrobial-resistant bacteria in developing countries [14].

Due to the rapid emergence of AMR and a dwindling antibiotics pipeline (principally, those drugs that would be useful in primary care) [15], the therapeutic armamentarium of physicians has narrowed considerably [16,17]. In fact, in 2011, the World Health Organization (WHO) decided on AMR (Combat Antimicrobial Resistance: No Action Today, No Cure Tomorrow) as the theme for World Health Day (7th of April), signifying the magnitude of this phenomenon and as being one of top three threats to humanity [18]. In addition to this, the European Centers for Disease Control and Prevention (ECDC) and the WHO have both introduced educational programs aimed at the public: the European Antibiotic Awareness Day on the 18th of November (since 2008), and the World Antimicrobial Awareness week (since 2015) [19]. The issues that are associated with SMA are particularly complex in the developing world, such as scarcity and poor quality of healthcare facilities at the primary care centers, lack of (official) access to medicines, and lack of strict policies for the regulation of the sales of medicines, predominantly antibiotics [20]. Furthermore, other critical problems related to the poor quality of healthcare services also exist, for example, long waiting times, difficulties in transportation and reaching the healthcare facilities, non-professional behavior of healthcare practitioners, presence of informal healthcare providers (without official training) and individuals not having healthcare insurance [21,22]. All these issues encourage the non-prescription use of antibiotics without medical consultation, and this behavior may ultimately worsen the AMR situation [23]. According to the 2018 WHO Report on surveillance on antibiotic consumption, disease-causing microorganisms are becoming more resistant against antibiotics, and this situation has been intensified by the uncontrolled use of antibacterial drugs. The report also suggests that systematic data for antimicrobial resistance from south-eastern Asian countries are lacking [24]. Based on the recent report published for the year 2019, around 4.95 million (3.62–6.57; 95% CI) deaths were associated with bacterial AMR, while 1.27 million (0.91–1.71; 95% CI) deaths directly attributable to AMR were reported worldwide [25].

By law, antibiotics may only be obtained from pharmacies in Pakistan through a prescription from a registered medical practitioner [26,27]. However, studies performed previously in Pakistan reported that people were obtaining antibiotics without prescription [26,28,29]. These studies were conducted long ago, and were unable to pinpoint which sociodemographic factors were associated with SMA practices. Thus, it is necessary to conduct a study that may help government authorities formulate strict policies to regulate pharmacies/medical stores not to sell antibiotics without prescriptions (over-the-counter; OTC) and help guide the preparation of educational interventions for the appropriate use of antibiotics, both for healthcare professionals and the public [30]. With this in mind, the present study aims to evaluate population knowledge, factors that may lead to SMA, common reasons related to SMA, the pattern of SMA, and sociodemographics associated with this practice in Pakistan.

## 2. Results

### 2.1. Sociodemographic Characteristics of Study Participants

Overall, a total of *n* = 480 study participants were included in this study; the demographic characteristics of the respondents are summarized in Table 1. In the study population, males were in the majority (55.6%; *n* = 267), and the mean age of the participants was 37.1 ± 10.1 years (median 36; range 21–70). The majority of the participants were in the age group of 31–40 years (44.2%; *n* = 212), and 76.3% (*n* = 366) of them were married. Most of the participants were self-employed (29.8%; *n* = 143) and had an undergraduate or bachelor’s degree (35.4%; *n* = 170). At the same time, 89% (*n*= 427) of participants had an income over PKR 20,000.

Among participants, 28.5% (*n* = 137) indicated that they had used antibiotics in the last month, 26.9% (*n* = 129) of participants stated that they had used antibiotics at least once in the previous six months, 14.4% (*n* = 69) used antibiotics in the previous 12 months, and 6% (*n* = 29) of the participants used antibiotics more than a year ago. At the same time, 22.9% (*n* = 110) of participants said that they do not remember when they used antibiotics the last time. In contrast, 75.8% (*n* = 364) of participants claimed that they had used antibiotics at least once at the time of data collection. Furthermore, 23.5% (*n* = 113) of participants claimed that they received advice from a doctor, pharmacist, or nurse on how to take these medicines, 40.5% (*n* = 194) indicated that they did not receive any advice from any healthcare professional, and 36.0% (*n* = 173) had no recollection on the topic.

### 2.2. Reliability of the Instrument

#### 2.2.1. Internal Consistency

Internal consistency (Cronbach’s α value) for the different domains of the instrument was determined: for the “Practices towards SMA” domain, the Cronbach’s α value was 0.817, while for the “Public-reported outcomes” domain, the α value was 0.658, respectively.

#### 2.2.2. Test–Retest Reliability

Results obtained from the test–retest analysis indicate satisfactory reliability and stability: for the “Practices towards SMA” domain and the “Public-reported outcomes” domain, the reliability coefficients were 0.972 and 0.861, respectively (*p* < 0.05).

### 2.3. Practices and Knowledge about SMA

#### 2.3.1. Prevalence of SMA among Study Participants

The total prevalence of SMA among study participants was 32.5%. Out of the 480 participants, 34.2% had used prescription antibiotics; 32.5% had self-medicated with antibiotics; and 33.3% did not remember whether they had used prescribed or non-prescribed antibiotics. The prevalence of SMA was higher in males (21.9%) compared to females (10.6%). Regarding age groups, the highest prevalence was noted among age groups of 31–40 years old (15.4%). On the other hand, the lowest prevalence of SMA was noted in the younger age group, 21–25 years old, where only 2.1% of participants had used antibiotics without a prescription. Postgraduate participants (2.9%) were less likely to use antibiotics than others. There was no significant correlation between SMA and some sociodemographic variables including age groups (*p* = 0.129), educational attainment (*p* = 0.224), income levels (*p* = 0.578,) and having insurance (*p* = 0.686). On the other hand, gender (*p* = 0.001), marital status (*p* = 0.003), occupation type (*p* = 0.001), and parents having children (*p* = 0.001) were significantly associated with SMA. All the results regarding SMA and associated demographic characteristics are shown in Table 2.

#### 2.3.2. Commons Reasons for SMA and Common Sources for Obtaining Antibiotics

**Figure 1:**
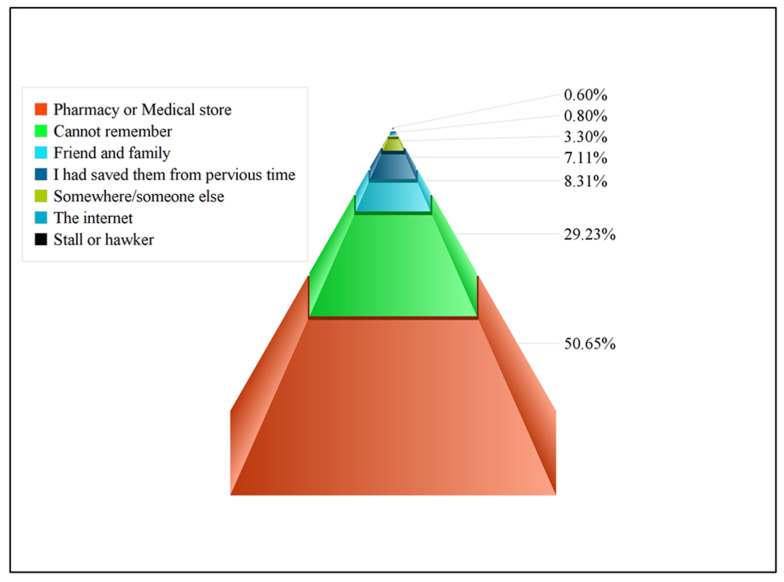
Common sources for obtaining antibiotics in the present study.

Results showed that most of the participants (35.4%) did not see doctors when they were sick because of lack of time. At the same time, 8.1% said that they do not implicitly trust doctors, and 15.2% of participants said that they do not have enough money for a doctor’s visit. In addition to this, 2.9% of participants stated that they face difficulties in communication with doctors, and 16.9% said that they face difficulties in reaching public health clinics or hospitals, or no transportation is available. The majority of participants who used antibiotics for themselves obtained their antibiotics from community pharmacies (50.6%), while 8.3% of participants obtained their antibiotics from a friend or family member. However, only (0.6%) of participants obtained their antibiotics from a stall or hawkers (Figure 1).

#### 2.3.3. Trends in Antibiotic Usage

**Figure 2:**
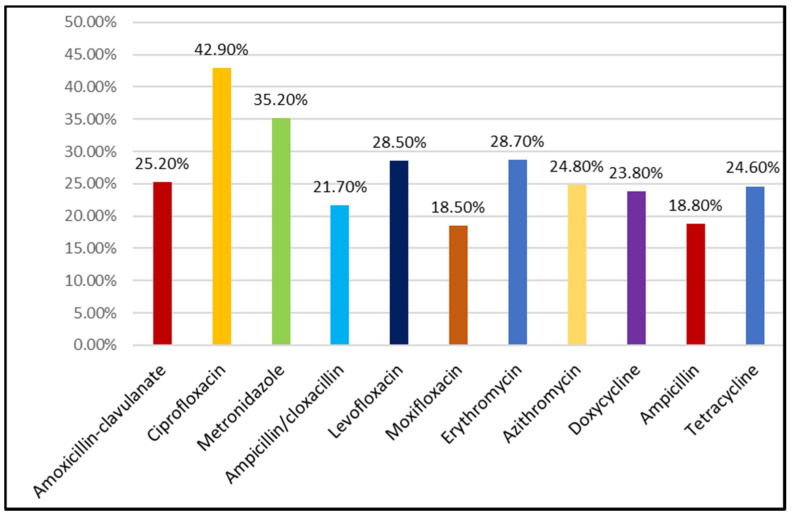
Most commonly used antibiotics for SMA in the study.

When participants were asked about why they consumed antibiotics; the respondents in the study referred to the following reasons for SMA: a cough or a cold, runny nose, a flu or sore throat, diarrhea or fever. The most commonly used antibiotic was ciprofloxacin (42.9%), followed by metronidazole (35.2%), erythromycin (28.7%), levofloxacin (28.5%), and amoxicillin-clavulanate (25.2%). The rest of the antibiotic groups inquired were less frequently utilized by study participants, depicting the frequency of usage for each antibiotic (Figure 2). Participants were allowed to choose more than one option, so the overall percentage is greater than 100%.

#### 2.3.4. Knowledge, Attitude, and Practices towards SMA

Less than half (41.7%) of participants completed their course of treatment as recommended, and 43.1% stopped taking their medicine when their symptoms began to improve. At the same time, 41.3% of participants indicated that it is okay to use antibiotics given to a friend or a family member, if they were used to treat the same illness, regardless of the outcome. More than half of the study participants (62.5%) wrongly identified that antibiotics are useful to treat colds and the flu (i.e., common viral infections). In addition, 67.7% believed that antibiotics may be used to treat fever followed by sore throat (50.4%), body aches (44.0%), headaches (42.7%) and urinary tract infections (UTIs) (40.2%) (Table 3).

#### 2.3.5. Predictors of SMA

Male gender (adjusted OR = 0.620; 95% CI: 0.383, 1.005), being in the age group of 31–40 years (adjusted OR = 0.550; 95%CI: 0.317, 0.953), and low education levels (primary school education [adjusted OR = 0.271; 95%CI: 0.101, 0.730] and secondary school education [adjusted OR = 0.325; 95%CI: 0.470, 0.719], certificate/diploma [adjusted OR = 0.250; 95%CI: 0.961, 0.649]) were more likely to predict self-medication use of antimicrobials. All the multivariate logistic regression analysis results of SMA covariates are presented in Table 4.

#### 2.3.6. Public reported outcomes

The last part of the study was specially designed to assess public perception after taking antibiotics. When participants of the study were asked whether they intend on self-medicating with antibiotics again if the same symptoms appear in the future, 41.8% gave a positive reply (i.e., they would do it again), while only 26.3% said that they will not self-medicate in the future; 31.9% of participants were unsure about their answer. A higher potential future SMA prevalence was noted among male participants (28.7% vs. 13.1%). The sociodemographic factors that were significantly associated with future prevalence of SMA were male gender (*p* ≤ 0.001), income rate (*p* ≤ 0.001), and occupation type (*p* = 0.003) (Table 5).

Moreover, Table 5 further includes results related to public reported outcomes. When participants were asked that how they perceived their symptoms after taking antibiotics, 72.9% of participants indicated that their symptoms improved after taking antibiotics. In contrast, 4.6% of participants stated that their symptoms worsened, and for 22.5% symptoms remained the same. At the same time, 45.8% of participants indicated that this practice of treating with antibiotics to take care of their health is acceptable, while 35.0% of participants did not agree with this statement. A total of 38.3% of participants stated that treating with antibiotics proved economical; 18.3% of participants were not sure about this. Among study participants, 20.4% indicated that they saved below PKR 500 on average at a time when they self-medicated with antibiotics, 10.9% of participants stated that they saved over PKR 1500, while 31.8% of participants could not say how much money they saved at one time.

## 3. Discussion

To the best of our knowledge, this is the first public-based study conducted in Islamabad, Pakistan to evaluate knowledge about antibiotics tendencies of self-medication with these drugs. Results showed that the prevalence of SMA was 32.5%, and the result was comparable with the study performed in the United Arab Emirates (UAE) (31.7%) [31]. In comparison, previous studies conducted in two different cities of Pakistan (Karachi, 80%; Peshawar, 69%) showed a higher prevalence of SMA than the present study [4,28]. In addition to this, studies performed in other developing countries, such as Kenya (60.0%), Nigeria (82.2%), Yemen (87.1%), and Saudi Arabia (40.8%) [32,33,34,35] also showed a higher prevalence of SMA by the public. The differences in the SMA rates may be due to ethnic diversity among the different populations and different healthcare systems in each of these countries. However, SMA rate may be decreased by adopting universal health coverage (UHC). UHC has also been adopted as a key target under the sustainable development goals by the United Nations, G20, and WHO to reduce AMR by improving the health system in terms of equity, quality, efficiency, accountability, sustainability and resilience [36]. Moreover, the present study also reported that male gender was an important predictor of SMA. Studies carried out in Pakistan and the UAE also showed that the prevalence of SMA was found to be higher among men [37], which might be due to men having greater access to medical stores and pharmacies in Pakistan. In comparison, other studies also showed similar results that women tended to use more antibiotics than men. Meanwhile, females are more prone to SMA, and also they visit physicians more often [38,39].

Results from the present study also indicated that most of the participants who self-medicated with antibiotics had a low level of education. A similar result was also achieved in studies carried out in Lebanon [29,40]. At the same time, in the present study, age was not linked to SMA, which is in contrast to other studies that have assigned either the young adult or adult population to be more prone towards practicing SMA; these studies were performed in Italy, Saudi Arabia, and United Arab Emirates [37,41,42]. Moreover, gender (*p* = 0.001), marital status (*p* = 0.003), occupation (*p* = 0.001), and parents having children (*p* = 0.001) were the factors that were significantly associated with SMA. Whereas findings from multivariate logistic analysis also found that male gender, being between 31 and 40 years of age with a low level of education are most prone towards SMA. A study performed in Malaysia and Uganda also confirmed the latter findings [43,44].

Another contributing factor is the lack of strict disciplinary regulation policies (compared to some Western countries), and due to this reason, people easily obtain antibiotics from pharmacies or medical stores without a prescription. The present study revealed that most of the participants obtained their antibiotics from pharmacies, either from a pharmacist or a pharmacy technician/assistant (50.6%). Results obtained from other studies performed in Malaysia, the UAE, and Pakistan also highlighted pharmacies as important sources of antibiotics for SMA [4,31,43]. A systematic review also provided evidence that community pharmacies remain the leading source of antibiotics for SMA in developing countries. This might be due to the profit-oriented nature of service delivery in this sector and/or inadequate supervision [22]. The present study also revealed that ciprofloxacin, followed by metronidazole and levofloxacin, were the most frequently used antibiotics for self-medication of cough, cold, flu, diarrhea, and fever. A study performed in Pakistan also showed similar results [29]. In contrast, other studies conducted in Pakistan showed that amoxicillin and metronidazole were the most commonly used antibiotics [4,45]. Interestingly, a scoping review noted that β-lactam antibiotics (more specifically, penicillin and aminopenicillin/β-lactamase inhibitors) are the most commonly used antimicrobials for SMA [46]. Other studies also indicated that antibiotics were most commonly used for fever, cold, and pain [41,47,48]. This irregular use of antibiotics for common viral infections such as cold and flu is an important factor in worsening antibiotic resistance; this serious problem was also verified by other studies [31,49,50].

In our study, it was noted that 40.2% of participants were most likely to stop taking antibiotics after they were feeling better. These results are similar to another study performed in Pakistan, where participants also stopped taking antibiotics on the third day or even earlier, if their symptoms improved [29]. This non-compliance may also lead to the spread of severely drug-resistant pathogens; according to the WHO, when any individual or patient stops taking antibiotics too early, it may benefit bacteria strains with some natural intrinsic resistance. As per the guidelines of the WHO, it is therefore advisable that individuals/patients always take the entire course of antibiotics prescribed to them, overseen by a certified healthcare professional (physicians, pharmacists, nurses or others). Moreover, the present study also revealed that the main reasons that influence the use of antibiotics for self-medication were the lack of trust in doctors and economic conditions; this was in accordance with another study [4]. The last part of the study consisted of public reported outcomes associated with SMA among our participants; 41.8% of participants indicated that they would still use antibiotics in the future if the same disease symptoms appear, and this rate is actually higher than the reported prevalence of SMA in the present population. This high future prevalence of SMA might be due to the high doctor’s fees for a checkup and poor patient management, as well as under-staffed healthcare centers that cannot bear the burden of the visiting population, eventually forcing people to look for alternative solutions and SMA seems to be the most viable choice. The future prevalence of SMA is significantly associated with the participant’s gender (*p* = 0.001), income levels (*p* = 0.001), and occupation type (*p* = 0.003). Moreover, most of the participants indicated that their symptoms improved after taking antibiotics, and 45.8% of participants said that treating with antibiotics is an acceptable practice.

This study has several limitations: as with any cross-sectional, questionnaire-based survey, there is no way to gauge the truthfulness of the answers from the participants. Furthermore, this study was conducted in a federal area (Islamabad); hence, these results cannot be generalized to the whole country.

## 4. Materials and Methods

### 4.1. Study Design and Settings

The present study adopted a prospective cross-sectional study design. This study was carried out in the capital city of Pakistan (Islamabad), located in the country’s northwest region. The total area of Islamabad is 906.5 km^2^, being the ninth largest city according to its population. The total population of Islamabad is above 2 million people, with an annual growth of 4.9% [51]. At the same time, the majority of the population in Islamabad city is 15–64 (59.38%) years old, while the literacy rate is also highest (88%) compared to other parts of the country [51].

### 4.2. Study Population

The participants included in this study were adults (≥18 years) willing to participate without having any communication problems either due to illness or other reasons. This study included laypeople only (i.e., people with no expert knowledge or professional qualifications in the particular subject) [52]. Participants with a medical background, such as doctors, pharmacists, nurses, paramedical and allied health professionals, were therefore excluded from this study. The respondents or participants were selected through convenience and snowball sampling methods. Furthermore, this study only included those participants who could read and write in Urdu and English.

### 4.3. Sample Size Calculation

To establish the required sample size for our study, a sample size calculation was performed by using Lorenz’s formula (1), described below [53]:

(1) $$n=z^{2}\frac{p\;x\;q}{d^{2}}$$

where *p* is the pre-study estimates of self-medication prevalence, *q* is 1 − *p*, *z* is 1.96 (standard normal deviation for a 95% CI), and *d* is 0.05 (tolerated sampling error or precision of 5%). At 95% CI, the *z* statistic value is 1.96, and *p* was determined to be 0.5 from a previous study, as reported by Bilal et al. [4]. Assuming a degree of precision of 0.05, the calculated minimum sample size was set at *n* = 234; however, to strengthen the study results, the final sample size was increased for contingency by more than 50%, and the final sample size of *n* = 480 persons was set.

### 4.4. Study Instrument

The research instrument utilized in the present study was a questionnaire containing both open-ended and close-ended (multiple choice) questions. Our research group developed the questionnaire, based on literature screening and screening the references of a scoping review conducted previously on the topic [46]. The questionnaire comprised 43 questions with a total of 71 items. The items in the questionnaire were broadly grouped into the following four categories: (i) sociodemographic characteristics of the participants (gender, age, marital status, education level, employment status, healthcare expense coverage); (ii) personal information on health and medicine use; (iii) practices towards SMA and factors contributing SMA; and (iv) public-reported outcomes. For the purpose of adaptation and translation of the questionnaire, a set criterion was followed, developed by Beaton et al. [54]. Before conducting the main study, pilot testing (involving *n* = 100 participants) of the translated questionnaires was carried out to ensure internal consistency (Cronbach alpha), reliability (test–retest reliability), face validity, and content validity. At the same time, pilot testing was necessary to determine if the questionnaire would be comprehensive, understandable, and appropriate among respondents [55]. The results obtained from the pilot testing of the questionnaire allowed quality improvement in view of specific cultural considerations, and thus, certain modifications were made in the wording of the questionnaire to produce the final instrument.

### 4.5. Data Collection

The data were collected in public places, including public parks, outside of big hospitals, shopping malls, bus stations, supermarkets, and metro stations. The final version of the questionnaire was used to collect data through the interview-administered method from February and July 2020. A written signed informed consent form was obtained from all participants who agreed to take part in this study. At the same time, an introductory letter was given to all respondents, which included a brief description of the study objectives and its importance. The data were collected through an interview-administered questionnaire by the principal investigator with the help of trained interviewers. The confidentiality and anonymity of the participants were protected throughout the study.

### 4.6. Statistical Analysis

The descriptive and inferential statistical data analyses were processed with the assistance of Statistical Package for the Social Sciences (SPSS) version 24 (SPSS Inc., Chicago, IL, USA). Descriptive statistics, including cross tables and frequencies, were used for variables, and a χ^2^ test was performed to determine variables associated with dependent variables. Multivariate logistic regression analysis was used to determine the predictors of SMA within the study population, and in this analysis, the prevalence of SMA was considered as the outcome. Participants’ sociodemographics were considered exposure variables (gender, age, income, education, job type, and health insurance availability). Finally, univariate and multivariate logistic regression at 95% CI were computed. All factors that achieved a *p*-value of less than 0.05 at the univariate level were considered fitting to be included in the multivariable model to determine the predictors of SMA. Statistical significance for all analyses was set at *p* < 0.05.

### 4.7. Ethical Considerations

Before the commencement of this study, ethical approval for this study was obtained from the ethical committee at International Islamic University Malaysia (IREC 2019-004) and Hamdard University Pakistan (HU-ERC-19-408).

## 5. Conclusions

In conclusion, SMA is common among the public of Pakistan; males were highlighted among the population to have higher intent for such use of these drugs. Underlying factors of why the public self-medicates with antibiotics were: mainly lack of trust towards doctors, economic considerations and easy availability of antibiotics as OTC from pharmacies. The lack of strict legislation/laws over the availability of antibiotics without prescription and limited availability of health insurance further complicates the issue. At the same time, healthcare professionals should play an important role in patient education, counseling and guide proper pharmaceutical use. Adequate competence and communication on the part of the healthcare professionals may increase patient trust towards them, which will—in turn—lead to higher rates of appropriate medicine use. The government must introduce educational and awareness programs specifically targeting the public so that people without sufficient knowledge are made aware of the dangers of SMA. Lastly, it is recommended to bring in legislation on managing antimicrobial prescriptions.

## Figures and Tables

**Table 1:** Sociodemographic characteristics of the participants included in the study.

Variables	Frequency (*n*)	Percentage (%)
Gender		
Male	267	55.6%
Female	213	44.4%
**Age (Years)**		
21–25 yrs	44	9.2%
26–30 yrs	95	19.8%
31–40 yrs	212	44.2%
Above 40 yrs	129	26.8%
**Marital status**		
Single	114	23.7%
Married	366	76.3%
**Education**		
Primary school	56	11.7%
Secondary school	124	25.8%
Certificate/Diploma	56	11.7%
Undergraduate degree/Bachelor’s degree	170	35.4%
Postgraduate education	74	15.4%
**Income**		
Under PKR 10,000	15	3.1%
PKR 10,000 to 20,000	38	7.9%
Over PKR 20,000	427	89%
**Occupation**		
Professional	94	19.6%
Skilled labor	21	4.4%
Manual labor	19	4.0%
Administrative	76	15.7%
Self-employed	143	29.8%
Home duties	93	19.4%
Unemployed	23	4.8%
Pensioner	11	2.3%
**Children**		
No	147	30.6%
Yes	333	69.4%
**Health Insurance**		
Yes	29	6%
No	434	90.4%
Not Sure	17	3.6%

**Table 2:** Prevalence of SMA and associated demographic characteristics.

Characteristics	Prevalence of SMA			*p*-Value
Gender	Prescription Antibiotics (*n*, %)	Non-Prescription Antibiotics (*n*, %)	Cannot Remember (*n*, %)	
Male	90 (18.8%)	105 (21.9%)	72 (15%)	0.001 ***
Female	74 (15.4%)	51 (10.6%)	88 (18.3%)	
**Age (years)**				
21–25 yrs	12 (2.5%)	10 (2.1%)	22 (4.6%)	0.129
26–30 yrs	33 (6.9%)	31 (6.5%)	31 (6.5%)	
31–40 yrs	64 (13.3%)	74 (15.4%)	74 (15.4%)	
Above 40 yrs	41 (8.5%)	55 (11.5%)	33 (6.8%)	
**Marital status**				
Married	128 (26.7%)	130 (27.1%)	108 (22.5%)	0.003 **
Single	36 (7.5%)	26 (5.4%)	52 (10.8%)	
**Education**				
Primary school	17 (3.5%)	23 (4.8%)	16 (3.3%)	0.224
Secondary school	37 (7.7%)	48 (10%)	39 (8.1%)	
Certificate/Diploma	13 (2.7%)	21 (4.4%)	22 (4.6%)	
Undergraduate degree	63 (13.1%)	50 (10.4%)	57 (11.9%)	
Postgraduate education	34 (7.1%)	14 (2.9%)	26 (5.5%)	
**Income**				
Under PKR 10,000	4 (0.8%)	4 (0.8%)	7 (1.5%)	0.578
PKR 10,000 to 20,000	11 (2.3%)	12 (2.5%)	15 (3.1%)	
Over PKR 20,000	149 (31.1%)	140 (29.2%)	138 (28.7%)	
**Occupation**				
Professional	43 (9%)	23 (4.8%)	28 (5.8%)	0.001 **
Skilled labor	3 (0.6%)	13 (2.7%)	5 (1%)	
Manual labor	3 (0.6%)	9 (1.9%)	7 (1.5%)	
Administrative	23 (4.8%)	27 (5.6%)	26 (5.4%)	
Self-employed	55 (11.9%)	53 (11%)	35 (7.3%)	
Home duties	29 (6%)	27 (5.6%)	37 (7.7%)	
Unemployed	3 (0.6%)	4 (0.8%)	16 (3.3%)	
Pensioner	5 (1%)	Nil	6 (1.3%)	
**Children**				
Yes	118 (24.5%)	122 (25.4%)	93 (19.4%)	0.001 ***
No	46 (9.6%)	34 (7.1%)	67 (14%)	
**Insurance**				
Yes	7 (1.5%)	15 (3.1%)	7 (1.5%)	0.686
No	145 (30.2%)	144 (30%)	145 (30.2%)	
Not sure	4 (0.8%)	5 (1%)	8 (1.7%)	

Legend: *** = *p* < 0.001, ** = *p* < 0.01.

**Table 3:** The participants’ responses regarding the knowledge and attitude questions.

Questions	Frequency (*n*)	Percentage (%)
Q. When do you think you should stop taking antibiotics once you have begun treatment?		
When I feel better	207	43.1%
When I have taken all the antibiotics as directed	200	41.7%
Do not know/Unsure of the answer	73	15.2%
Q. It is okay to use antibiotics that were given to you by a friend or family member if they were used to treat the same illness?		
Yes	198	41.3%
No	168	35.0%
Do not know	114	23.7%
Q. It is okay to buy the same antibiotics, or request the same antibiotics from a doctor if I am sick, and they helped me get better when I had the same symptoms before?		
Yes	256	53.3%
No	120	25.0%
Do not know	104	21.7%
Q. Do you think that the above mentioned conditions may be treated with antibiotics? (Mark more than one if you deem appropriate)		
HIV/AIDS	107	22.3%
Gonorrhea	137	28.5%
Bladder infection or UTI	193	40.2%
Diarrhea	171	35.6%
Cold and Flu	300	62.5%
Fever	325	67.7%
Malaria	114	23.8%
Measles	97	20.2%
Skin or wound infection	148	30.8%
Sore throat	242	50.4%
Body aches	211	44%
Headaches	205	42.7%

**Table 4:** Multivariate logistic regression analysis of predictors for SMA.

Variable	COR (CI 95%)	*p*-Value	AOR (CI 95%)	*p*-value
**Gender**				
Male	0.591 (0.375–0.931)	0.023 *	0.620 (0.383–1.005)	0.052
Female	Ref		Ref	
**Income**				
Below PKR 10,000	0.940 (0.231–3.829)	0.940	1.319 (0.301–5.780)	0.713
PKR 10,000–20,000	0.861 (0.368–2.015)	0.051	2.657 (0.515–3.345)	0.589
Above PKR 20,000	Ref		Ref	
**Health insurance**				
No	1.259 (0.331–4.782)	0.736	1.174 (0.299–4.616)	0.818
Yes	2.679 (0.545–13.157)	0.025 *	2.657 (0.515–13.696)	0.243
Not sure	Ref		Ref	
**Age**				
21–25 years	0.895 (0352–2.271)	0.815	0.552 (0.207–1.472)	0.235
26–30 years	0.794 (0.420–1.498)	0.794	0.555 (0.281–1.096)	0.090
31–40 years	0.645 (0.381–1.090)	0.050	0.550 (0.317–0.953)	0.033 *
Above 40 years	Ref	Ref	Ref	Ref
**Education**				
Primary school	0.304 (0.126–0.736)	0.008	0.271 (0.101–0.730)	0.010 *
Secondary school	0.317 (0.149–0.676)	0.003	0.325 (0.470.719)	0.005 **
Certificate/Diploma	0.255 (0.101–0.646)	0.004	0.250 (0.961–0.649)	0.004 **
Undergraduate degree	0.519 (0.251–1.071)	0.076	0.521 (0.250–1.087)	0.082
Postgraduate education	Ref		Ref	

Legend: ** = *p* < 0.01; * = *p* < 0.05; AOR = Adjusted Odds Ratio; CI = Confidence Interval; COR = Crude Odds Ratio; *Ref* = Reference category.

**Table 5:** Public reported outcomes for the participants in the study.

Demographic Characteristics	Statement Do You Intend to Self-Medicate again if the Same Symptoms Appear in the Future?			*p*-Value
	Yes	No	Not Sure	
**Gender**				
Male	138 (28.7%)	57 (11.9%)	72 (15%)	0.001 ***
Female	63 (13.1%)	69 (14.4%)	81 (16.9%)	
**Income**				
Under PKR 10,000	9 (1.9%)	2 (0.4%)	4 (0.8%)	0.001 ***
PKR 10,000 to 20,000	26 (5.4%	9 (1.9%)	3 (0.6%)	
Over PKR 20,000	166 (34.6%)	115 (24%)	146 (30.4%)	
**Occupation**				
Professional	28 (5.8%)	26 (5.4%)	40 (8.3%)	0.003 **
Skilled labor	12 (2.5%)	5 (1%)	4 (0.8%)	
Manual labor	13 (2.7%)	5 (1%)	1 (0.2%)	
Administrative	28 (5.8%)	26 (5.4%)	22 (4.6%)	
Self-employed	67 (14%)	30 (6.3%)	46 (9.6%)	
Home duties	35 (7.3%)	24 (5%)	34 (7.1%)	
Unemployed	12 (2.5%)	8 (1.7%)	3 (0.6%)	
Pensioner	6 (1.4%)	2 (0.4%)	3 (0.6%)	
**How do you evaluate your symptoms after taking antibiotics?**				
**Better**	**Worse**		**Same as before**	
350 (72.9%)	22 (4.6%)		108 (22.5%)	
**What do you think about practicing SMA to take care of your health?**				
**Good practice**	**Acceptable practice**		**Not acceptable practice**	
92 (19.2%)	220 (45.8%)		168 (35%)	
**Do you feel that treating your symptoms with antibiotics proves to be economical?**				
**Yes, it’s economical**	**I cannot say**	**Not sure**	**Don’t know at all**	
184 (38.3%)	130 (27.1%)	88 (18.3%)	78 (16.3%)	
**On average, how much money (PKR) did you save one time when you self-medicate with antibiotics?**				
**Under PKR 500**	**PKR 600 to 1000**	**PKR 1000 to 1500**	**Above PKR 1500**	**I cannot say**
98 (20.4%)	107 (22.3%)	70 (14.6%)	52 (10.9%)	153 (31.8%)

Legend: *** = *p* < 0.001; ** = *p* < 0.01.

## Data Availability

The anonymized datasets used and/or analyzed during the present study are available from the corresponding author on reasonable request.

## References

[1] Guinovart M.C., Figueras A., Llop J.C., Llor C. (2015). Obtaining antibiotics without prescription in Spain in 2014: Even easier now than 6 years ago. J. Antimicrob. Chemother..

[2] Widayati A., Suryawati S., de Crespigny C., Hiller J.E. (2011). Self medication with antibiotics in Yogyakarta City Indonesia: A cross sectional population-based survey. BMC Res. Notes.

[3] Alghadeer S., Aljuaydi K., Babelghaith S., Alhammad A., Alarifi M.N. (2018). Self-medication with antibiotics in Saudi Arabia. Saudi Pharm. J..

[4] Bilal M., Haseeb A., Khan M.H., Arshad M.H., Ladak A.A., Niazi S.K., Musharraf M.D., Manji A.A. (2016). Self-Medication with Antibiotics among People Dwelling in Rural Areas of Sindh. J. Clin. Diagn. Res. JCDR.

[5] Llor C., Bjerrum L. (2014). Antimicrobial resistance: Risk associated with antibiotic overuse and initiatives to reduce the problem. Ther. Adv. Drug Saf..

[6] European Commission Antibiotics Resistance. Survey Requested by the European Commission, Directorate-General for Health and Food Safety and Co-Ordinated by the Directorate-General for Communication. https://www.eusaferhealthcare.eu/wp-content/uploads/ebs_478_en-1-min.pdf.

[7] WHO Antibiotic Resistance: Multi-Country Public Awareness Survey. World Health Organization. https://apps.who.int/iris/handle/10665/194460.

[8] Panasiuk L., Lukas W., Paprzycki P., Verheij T., Godycki-Ćwirko M., Chlabicz S. (2010). Antibiotics in the treatment of upper respiratory tract infections in Poland. Is there any improvement?. J. Clin. Pharm. Ther..

[9] Zajmi D., Berisha M., Begolli I., Hoxha R., Mehmeti R., Mulliqi-Osmani G., Kurti A., Loku A., Raka L. (2017). Public knowledge, attitudes and practices regarding antibiotic use in Kosovo. Pharm. Pract..

[10] Janssen J., Afari-Asiedu S., Monnier A., Abdulai M.A., Tawiah T., Wertheim H., Baltussen R., Asante K.P. (2022). Exploring the economic impact of inappropriate antibiotic use: The case of upper respiratory tract infections in Ghana. Antimicrob. Resist. Infect. Control.

[11] Rijal K.R., Banjara M.R., Dhungel B., Kafle S., Gautam K., Ghimire B., Ghimire P., Dhungel S., Adhikari N., Shrestha U.T. (2021). Use of antimicrobials and antimicrobial resistance in Nepal: A nationwide survey. Sci. Rep..

[12] Gajdács M., Urbán E., Stájer A., Baráth Z. (2021). Antimicrobial Resistance in the Context of the Sustainable Development Goals: A Brief Review. Eur. J. Investig. Health Psychol. Educ..

[13] WHO International Organizations Unite on Critical Recommendations to Combat Drug-Resistant Infections and Prevent Staggering Number of Deaths Each Year. https://www.who.int/news/item/29-04-2019-new-report-calls-for-urgent-action-to-avert-antimicrobial-resistance-crisis.

[14] O’Neill J. Review on Antimicrobial Resistance, December 2014. http://amr-review.org/.

[15] Gajdács M. (2019). The concept of an ideal antibiotic: Implications for drug design. Molecules.

[16] Laxminarayan R., Duse A., Wattal C., Zaidi A.K., Wertheim H.F., Sumpradit N., Vlieghe E., Hara G.L., Gould I.M., Goossens H. (2013). Antibiotic resistance-the need for global solutions. Lancet Infect. Dis..

[17] Chang H.H., Cohen T., Grad Y.H., Hanage W.P., O’Brien T.F., Lipsitch M. (2015). Origin and proliferation of multiple-drug resistance in bacterial pathogens. Microbiol. Mol. Biol. Rev..

[18] WHO Antimicrobial Resistance: No Action Today, No Cure Tomorrow. https://www.who.int/dg/speeches/2011/WHD_20110407/en/.

[19] Earnshaw S., Monnet D.L., Duncan B., O’Toole J., Ekdahl K., Goossens H. (2009). European Antibiotic Awareness Day, 2008—The first Europe-wide public information campaign on prudent antibiotic use: Methods and survey of activities in participating countries. Eurosurveillance.

[20] Aslam A., Gajdács M., Zin C.S., Ab Rahman N.S., Ahmed S.I., Zafar M.Z., Jamshed S. (2020). Evidence of the Practice of Self-Medication with Antibiotics among the Lay Public in Low- and Middle-Income Countries: A Scoping Review. Antibiotics.

[21] Hadi U., Duerink D.O., Lestari E.S., Nagelkerke N.J., Werter S., Keuter M., Suwandojo E., Rahardjo E., van den Broek P., Gyssens I.C. (2008). Survey of antibiotic use of individuals visiting public healthcare facilities in Indonesia. Int. J. Infect. Dis..

[22] Ocan M., Obuku E.A., Bwanga F., Akena D., Richard S., Ogwal-Okeng J., Obua C. (2015). Household antimicrobial self-medication: A systematic review and meta-analysis of the burden, risk factors and outcomes in developing countries. BMC Public Health.

[23] Uddin T.M., Chakraborty A.J., Khusro A., Zidan B.R.M., Mitra S., Emran T.B., Dhama K., Ripon M.K.H., Gajdács M., Sahibzada M.U.K. (2021). Antibiotic resistance in microbes: History, mechanisms, therapeutic strategies and future prospects. J. Infect. Public Health.

[24] WHO (2018). Report on Surveillance of Antibiotic Consumption. https://www.who.int/medicines/areas/rational_use/who-amr-amc-report-20181109.pdf.

[25] Murray C.J., Ikuta K.S., Sharara F., Swetschinski L., Aguilar G.R., Gray A., Han C., Bisignano C., Rao P., Wool E. (2022). Global burden of bacterial antimicrobial resistance in 2019: A systematic analysis. Lancet.

[26] Qidwai W., Krishanani M.K., Hashmi S., Afridi M., Ali R.A. (2006). Private drug sellers’ education in improving prescribing practices. J. Coll. Physicians Surg. Pak..

[27] Saleem Z., Hassali M.A., Godman B., Fatima M., Ahmad Z., Sajid A., Rehman I.U., Nadeem M.U., Javaid Z., Malik M. (2020). Sale of WHO AWaRe groups antibiotics without a prescription in Pakistan: A simulated client study. J. Pharm. Policy Pract..

[28] Khan S.J., Khan S., Shah N. (2011). Self-medication with antibiotics in urban areas of Peshawar. Gomal J. Med. Sci..

[29] Nazir S., Azim M. (2017). Assessment of antibiotic self-medication practice among public in the northwestern region of Pakistan. Eur. J. Hosp. Pharm..

[30] Michaelidou M., Karageorgos S.A., Tsioutis C. (2020). Antibiotic Use and Antibiotic Resistance: Public Awareness Survey in the Republic of Cyprus. Antibiotics.

[31] Abduelkarem A.R., Othman A.M., Abuelkhair Z.M., Ghazal M.M., Alzouobi S.B., El Zowalaty M.E. (2019). Prevalence Of Self-Medication with Antibiotics Among Residents in United Arab Emirates. Infect. Drug Resist..

[32] Nyambega J.O. (2017). Antibiotic use and misuse among adults in Magwagwa Ward, Nyamira County in Kenya. Age Ageing.

[33] Abdulraheem I., Adegboye A., Fatiregun A. (2016). Self-medication with antibiotics: Empirical evidence from a Nigerian rural population. Br. J. Pharm. Res..

[34] Albawani S.M., Hassan Y.B., Abd-Aziz N., Gnanasan S. (2017). Self-medication with antibiotics in Sana’a City, Yemen. Trop. J. Pharm. Res..

[35] Al-Qahtani M.A., Amin H.S., Al-Qahtani A.A., Alshahrani A.M., Alghamdi H.A., Althwayee M.S., Alzahrani A.A. (2018). Self-medication with Antibiotics in a primary care setting in King Khalid University Hospital, Riyadh, Saudi Arabia. J. Family Community Med..

[36] Bloom G., Merrett G.B., Wilkinson A., Lin V., Paulin S. (2017). Antimicrobial resistance and universal health coverage. BMJ Glob. Health.

[37] Abasaeed A., Vlcek J., Abuelkhair M., Kubena A. (2009). Self-medication with antibiotics by the community of Abu Dhabi Emirate, United Arab Emirates. J. Infect. Dev. Ctries..

[38] Grigoryan L., Burgerhof J.G., Degener J.E., Deschepper R., Lundborg C.S., Monnet D.L., Scicluna E.A., Birkin J., Haaijer-Ruskamp F.M. (2008). Determinants of self-medication with antibiotics in Europe: The impact of beliefs, country wealth and the healthcare system. J. Antimicrob. Chemother..

[39] Ramay B.M., Lambour P., Cerón A. (2015). Comparing antibiotic self-medication in two socio-economic groups in Guatemala City: A descriptive cross-sectional study. BMC Pharmacol. Toxicol..

[40] Jamhour A., El-Kheir A., Salameh P., Hanna P.A., Mansour H. (2017). Antibiotic knowledge and self-medication practices in a developing country: A cross-sectional study. Am. J. Infect. Control.

[41] Napolitano F., Izzo M.T., Di-Giuseppe G., Angelillo I.F. (2013). Public knowledge, attitudes, and experience regarding the use of antibiotics in Italy. PLoS ONE.

[42] Emeka P.M., Al-Omar M., Khan T.M. (2014). Public attitude and justification to purchase antibiotics in the Eastern region Al Ahsa of Saudi Arabia. Saudi Pharm. J..

[43] Aslam A., Zin C.S., Ab Rahman N.S., Gajdács M., Ahmed S.I., Jamshed S. (2021). Self-Medication Practices with Antibiotics and Associated Factors among the Public of Malaysia: A Cross-Sectional Study. Drug Healthc. Patient Saf. Surg..

[44] Ocan M., Bwanga F., Bbosa G.S., Bagenda D., Waako P., Ogwal-Okeng J., Obua C. (2014). Patterns and predictors of self-medication in northern Uganda. PLoS ONE.

[45] Malik U.R., Chang J., Hashmi F., Atif N., Basir H., Hayat K., Khan F.U., Kabba J.A., Lambojon K., Fang Y. (2021). A Simulated Client Exploration of Nonprescription Dispensing of Antibiotics at Drugstores for Pediatric Acute Diarrhea and Upper Respiratory Infection in Lahore, Pakistan. Infect. Drug Resist..

[46] Aslam A., Gajdács M., Zin C.S., Ab Rahman N.S., Ahmed S.I., Jamshed S. (2020). Public Awareness and Practices towards Self-Medication with Antibiotics among the Malaysian Population. A Development of Questionnaire and Pilot-Testing. Antibiotics.

[47] Shah S.J., Ahmad H., Rehan R.B., Najeeb S., Mumtaz M., Jilani M.H., Rabbani M.S., Alam M.Z., Farooq S., Kadir M.M. (2014). Self-medication with antibiotics among non-medical university students of Karachi: A cross-sectional study. BMC Pharmacol. Toxicol..

[48] Pan H., Cui B., Zhang D., Farrar J., Law F., Ba-Thein W. (2012). Prior knowledge, older age, and higher allowance are risk factors for self-medication with antibiotics among university students in southern China. PLoS ONE.

[49] Chanvatik S., Kosiyaporn H., Lekagul A., Kaewkhankhaeng W., Vongmongkol V., Thunyahan A., Tangcharoensathien V. (2019). Knowledge and use of antibiotics in Thailand: A 2017 national household survey. PLoS ONE.

[50] Al-Rasheed A., Yagoub U., Alkhashan H., Abdelhay O., Alawwad A., Al-Aboud A., Battal S.A. (2016). Prevalence and Predictors of Self-Medication with Antibiotics in Al Wazarat Health Center, Riyadh City, KSA. BioMed Res. Int..

[51] Pakistan, Govt Second Phase of Census District Wise. https://web.archive.org/web/20170829164748/http://www.pbscensus.gov.pk/sites/default/files/DISTRICT_WISE_CENSUS_RESULTS_CENSUS_2017.pdf.

[52] Dicitionary O. Oxford Learners Dictionary. https://www.oxfordlearnersdictionaries.com/definition/english/layperson?q=layperson.

[53] Charan J., Biswas T. (2013). How to calculate sample size for different study designs in medical research?. Indian J. Psychol. Med..

[54] Beaton D.E., Bombardier C., Guillemin F., Ferraz M.B. (2000). Guidelines for the process of cross-cultural adaptation of self-report measures. Spine.

[55] Károlyházy K., Fazekas B., Fazekas J., Hermann P., Márton K. (2016). Ebola virus disease: Awareness among dental students in Hungary. Acta Microbiol. Immunol. Hung..

